# P-1019. Comparative Analysis of C. difficile Isolates from Recurrent and Non-Recurrent Infections: Outcomes, Antimicrobial Susceptibility, and Biofilm Formation

**DOI:** 10.1093/ofid/ofaf695.1215

**Published:** 2026-01-11

**Authors:** Maria Luana G S Morais, Monica Josiane Rodrigues- Jesus, Elliot Euntaek Jang, Deiziane Costa, Cirle A Warren

**Affiliations:** University of Virginia, Charlottesville, VA; Univesity of Virginia, Charlottesville, Virginia; University of Virginia, Charlottesville, VA; University of Virginia, Charlottesville, VA; University of Virginia, Charlottesville, VA

## Abstract

**Background:**

*Clostridioides difficile* infection (CDI) is a serious diarrheal illness caused by microbiota disruption, mainly after antibiotic use. Around 20-25% of patients experience recurrence, and the risk increases for each episode. The causes of recurrence are not entirely elucidated. This study aimed to investigate differences in antibiotic susceptibility and biofilm formation between strains isolated from patients with recurrent and non-recurrent CDI.

**Methods:**

We collected fecal samples from adult patients > 18 years old and with diarrhea, with a positive GeneXpert-positive test for

*tcdB* Patient demographic and clinical data were collected from electronic medical records. *Clostridioides difficile* was isolated using alcohol shock treatment and cultured on selective TCCFA media under anaerobic conditions. Antimicrobial susceptibility assays (for Metronidazole, Tigecycline, Vancomycin, Omadacycline, Eravacycline) were assessed using e-test strips (Liofichem), with interpretation following CLSI guidelines. Biofilm biomass was quantified using the crystal violet staining technique.

**Results:**

54 *C. difficile* isolates were analyzed: 23 from R-CDI patients and 31 from NR-CDI patients. Antibiotic exposure within 14 days before hospitalization occurred in most patients with NR-CDI (91.30%) and R-CDI (79.47%). The predominant toxinotype was

*tcdA⁺tcdB⁺cdt-* for both R-CDI and NR-CDI patients. All isolates were 100% susceptible to metronidazole, vancomycin, omadacycline, eravacycline and tigecycline. Among R-CDI, MIC ranges were 0.25-4 mg/mL for metronidazole and 0.38-8 mg/mL for vancomycin. For NR-CDI, MIC ranges were 0.75-4 mg/mL for metronidazole and 0.5-4mg/mL for vancomycin. Biofilm formation was observed in most isolates, and they were categorized as low, moderate, or high producers, with maximum biofilm formation after 48h. The biofilm biomass was strain and time-dependent, but no significant differences between R-CDI and NR-CDI strains was observed at any of the time points analyzed.

**Conclusion:**

Our findings in this small study did not find significant differences in antimicrobial susceptibility, biofilm formation and microbiological characteristics among clinical isolates from recurrent and non-recurrent CDI.

**Disclosures:**

All Authors: No reported disclosures

